# The role of personality traits and social support in relations of health-related behaviours and depressive symptoms

**DOI:** 10.1186/s12888-022-03693-w

**Published:** 2022-01-22

**Authors:** Johanna-Sophie Edler, Kristin Manz, Natalia Rojas-Perilla, Harald Baumeister, Caroline Cohrdes

**Affiliations:** 1 Mental Health Research Unit, Department of Epidemiology and Health Monitoring, Robert Koch Institute, Unit 26 Mental Health, PO Box 650261, 13302 Berlin, Germany; 2 Physical Health Research Unit, Department of Epidemiology and Health Monitoring, Robert Koch Institute, Berlin, Germany; 3 Department of Analytics in the Digital Era, United Arab Emirates University, Al Ain, UAE; 4 Department of Clinical Psychology and Psychotherapy, Ulm University, Ulm, Germany

**Keywords:** Depression, Personality, Social support, Health-related behaviours, Public mental health, Mental health monitoring

## Abstract

**Background:**

Previous evidence has suggested that physically inactive individuals and extensive media users are at high risk for experiencing depressive symptoms. We examined personality traits and perceived social support as potential moderators of this association. Personality and perceived social support were included as two of the most frequently considered variables when determining predispositioning factors for media use phenomena also discussed in relation to physical activity.

**Methods:**

We analysed cross-sectional data from 1402 adults (18–31 years old) who participated in a national health survey in Germany (KiGGS, Study on the health of children and adolescents in Germany, wave 2). The data included one-week accelerometer assessments as objective indicators of physical activity, self-reported media use, depressive symptoms, perceived social support and Big 5 personality traits. An elastic net regression model was fit with depressive symptoms as outcome. Ten-fold cross-validation was implemented.

**Results:**

Amongst the main effects, we found that high media use was positively correlated with depressive symptoms, whereas physical activity was not correlated. Looking at support and individual differences as moderators, revealed that PC use was more strongly correlated with depressive symptoms in cases of low levels of perceived social support. Positive associations of social media use with depressive symptoms were more pronounced, whereas negative associations of moderate to vigorous physical activity with depressive symptoms were less pronounced in extraverts than they were in introverts.

**Conclusions:**

Results highlight the importance of considering individual factors for deriving more valid recommendations on protective health behaviours.

**Supplementary Information:**

The online version contains supplementary material available at 10.1186/s12888-022-03693-w.

## Introduction

Depression is amongst the most frequent diseases worldwide and is the main contributor of (nonfatal) morbidity [1]. In particular, young adults have high prevalence rates of depressive symptoms [2]. In addition to age, several other risk and protective factors for depressive symptoms have been identified, including health-related behaviours, that include risk and health protective behaviours [3–5]. Most health-related behaviours are modifiable, and behaviour change is an important component for preventing mental disorders [6]. Therefore, more detailed knowledge about the associations of depressive symptoms and specific health-related behaviours represents an essential starting point for public health research.

Current findings list extensive media use (e.g., high amounts of screen-time) and the lack of physical activity as major behaviour-related risk factors for depressive symptoms [7–9]. Both factors are also behavioural parameters that can be measured automatically via mobile devices and are thus effective and objective in terms of research economics (e.g., via accelerometers or smartphones). In light of their association with mental health, these factors are now also increasingly investigated by public health institutions; thus, validating the predictive accuracy of these health-related behaviours is relevant.

People who report extensive internet and social media use also more frequently report psychopathological symptoms [10].

In contrast to media use, physical activity has been reported to be negatively correlated with depressive symptoms and thus may serve as a protective factor [11]. Physical activity is defined as any bodily movement produced by skeletal muscles that requires energy expenditure [12]. The World Health Organization recommends at least 150 min of moderate-intensity physical activity or 75 min of vigorous-intensity physical activity per week for adults [13]. The lack of physical activity includes behaviour that involves a low level of energy expenditure while sitting or lying [13]. The present study focuses on media use and physical activity as important health-related behaviours affecting depressive symptoms.

To validate the relationship between health-related behaviours, such as media use and physical activity, and depressive symptoms, the individual differences must be taken into account [14–16]. Previous reports have proposed that associations of media use [17, 18] and physical activity [19, 20] with depressive symptoms are further qualified by individual-level factors such as personality and perceived social support.

To understand the role of personality traits, McCrae and Costa [21] pointed out the relevance of observing behavioural correlates. In line with this assumption, empirical evidence by Nagata and colleagues [22] showed how the correlation of physical activity with depressive symptoms differs after controlling for certain personality characteristics.

With regard to media use, Seidman [23] suggested that how individuals use and experience social media differs by personality, thus underpinning this assumption.

Despite these findings, many studies that have found relationships between high media use or a lack of physical activity and depressive symptoms did not examine the potential moderating function of personality [24].

Perceived level of social support [25] has been discussed as a major factor when investigating motivations and effects of media usage [26]; this variable may also function as a protective factor for mental health [27, 28]. Until now, evidence for the role of perceived social support on differences in associations between media use and depressive symptoms is still pending. Consequently, a profound understanding of the role of personality traits and perceived social support on the associations between health-related behaviours and depressive symptoms could be an important first step towards developing tailored prevention strategies.

### The roles of personality traits and perceived social support in associations between media use and depressive symptoms

Previous studies have shown inconsistent results regarding individuals’ susceptibility to media and have indicated differential effects depending on individual-level characteristics [24]. For example, small media effect sizes on mood have been explained with the selectivity of media use in accordance with the selective exposure theory [29]. The theory explains individuals’ tendency to search for content and information that match their personal needs and beliefs. Valkenburg and colleagues [24] complemented selective exposure theory by concluding that media choices may also depend on dispositional factors such as personality.

Personality psychology often refers to the five-factor personality model [21], which is comprised of the dimensions of extraversion, neuroticism, conscientiousness, agreeableness and openness. Extraversion can be described as “energetic and thrill-seeking versus sober and solitary”, neuroticism as “chronically predisposed to emotional distress versus emotionally stable”, conscientiousness as “disciplined and fastidious versus laidback and careless”, agreeableness as “kind and trusting versus competitive and arrogant” and openness as “curious and unconventional versus traditional and pragmatic” [30].

In fact, personality traits have been linked to different patterns of social media use in the past [31]. Valkenburg and colleagues [24] explained the differential effects of media use through cognitive, emotional and physiological processes that occur *during* media use. Individuals with low levels of self-esteem perceived more frequent social comparisons on the social media platform Facebook and compared themselves more often with others, which resulted in downward comparisons and self-devaluation [32]. Since low self-esteem has been related to the concept of neuroticism [33], emotionally labile people may more frequently experience downward comparisons and self-devaluation and hence have a more stressful user experience in social media compared with that of emotionally stable individuals. In sum, theories on the relationships between media use and depressive symptoms with a focus on personality are rare [34]. Following the summary of theories by Valkenburg and colleagues [24], another relevant individual-level factor that is related to the strength of media effects is social context. One aspect of social context is social support. Social support has been defined as the “social resources that persons perceive to be available or that are actually provided to them” [35]. This definition makes a crucial distinction: received support refers to retrospective reports about help received in the past. Perceived social support refers to a more or less stable expectation that help is available should the need arise. Although both forms of support are usually related, their association is only moderate in size, and they show distinct relations with indicators of physical and mental health [36]. In the present report, the potential moderating function of perceived social support was examined.

Seidman proposed that social media might be used to compensate for missing real-life social contacts [23]. Thus, when persons perceive themselves as receiving much support in real life, online compensation may be less sought after and likely less important for their mental well-being.

In conclusion, personality and perceived social support are important aspects of a thorough understanding of social media use and its relationship to mental health [23].

### The role of personality traits and perceived social support in associations between physical activity and depressive symptoms

Another, relatively larger, body of research has investigated differential associations of physical activity with depressive symptoms [15, 16, 37]. However, the understanding of the underlying factors determining these differential effects remains insufficient [38].

According to a review by Kandola and colleagues [38], the association of physical activity and depressive symptoms depends on biological and psychosocial mechanisms. Theories explaining the association of physical activity and depressive symptoms with biological mechanisms have described depressive symptoms as elevated levels of pro-inflammatory markers and as a consequence of high cortisol due to hypothalamic-pituitary-adrenal (HPA) axis dysregulation [39]. Moreover, chronic stress has been suggested as a mediating mechanism behind associations of inflammatory or neuroendocrinological reactions and depressive symptoms [40]. Accordingly, physical activity that has been found to be correlated with a reduction in these biological mechanisms [38] may lead to lower depressive symptoms [41]. The association of physical activity and reduced depressive symptoms is thought to be higher with higher levels of biological dysregulation as a result of chronic stress. Since emotionally labile individuals frequently report chronic social stress, they may benefit from physical activity in particular [30].

In addition to the perspective on the personality of the individual, Kandola and colleagues [38] further explained the association of physical activity and depressive symptoms with psychosocial mechanisms. These theories proposed associations of depressive symptoms with low levels of perceived social support [42] that are more often observed in emotionally labile individuals and are rarely observed in extraverts [33, 43, 44]. Conversely, physical activity has been found to be associated with opportunities to extend the social network and thereby increase perceptions of social support [38]. The association of physical activity and reduced depressive symptoms is assumed to be higher at lower levels of perceived social support. The latter is supposed to be found in emotionally labile individuals.

Previous research has shown that high levels of physical activity were correlated with decreased levels of depressive symptoms by reinforcing energy and enthusiasm [23]. Highly extraverted individuals show characteristics that are typically described as energetic and talkative, regardless of their physical activity level [23]. Hence, physical activity may be particularly helpful for introverts, but not for extraverts.

### The present study

The current study aimed to assess the relationship between health-related behaviours and depressive symptoms at the individual level.

Based on the theoretical and empirical background, we hypothesized that personality traits and perceived social support moderate the association between media use and depressive symptoms (hypothesis 1). We expected extraversion and perceived social support to buffer the positive association between high media use and depressive symptoms. By contrast, neuroticism was expected to increase the positive association between high media use and depressive symptoms.

In hypothesis 2, we assumed that personality traits and perceived social support moderate the association between physical activity and depressive symptoms. More precisely, we expected that extraversion and perceived social support would buffer the negative association between physical activity and depressive symptoms, whereas neuroticism would increase the negative association between physical activity and depressive symptoms.

The lack of prior theorizing and evidence does not allow us to form hypotheses on the moderating role of the personality dimensions of openness, conscientiousness and agreeableness in the associations between media use and physical activity with depressive symptoms. Thus, we explored their contributions to the prediction of depressive symptoms and their interaction with media use and physical activity in our analyses.

## Method

### Sample and procedure

This study used data from the second follow-up of the KiGGS cohort of the German Public Health Institute [45]. The KiGGS is a cross-sectional regularly conducted health interview and examination survey for children and adolescents that is combined with a longitudinal cohort sample [45]. Because of the longitudinal cohort structure, some of the study participants reached adulthood at the time of the second data collection. The current study analysed the self-report questionnaires that all participants had to complete. Additionally, the participants wore an accelerometer for 7 days. A detailed description of the recruitment procedure and study protocol can be found in the original publication by Mauz and colleagues [45, 46]. All the participants gave informed written consent for participation in the study. The second follow-up of the KiGGS survey was conducted in accordance with the amended Declaration of Helsinki and approved by the local independent Ethics Committee of the Medical University Hanover, Germany.

Since depressive symptoms were measured in individuals of full age only the original sample of *n* = 2873 is based on the adult participants of the KiGGS cohort in the second survey wave (KiGGS wave 2). Non-response (22.3%, *n* = 642), technical problems (16.0%, *n* = 358) or non-compliance (18.4%, *n* = 345) resulted in a sample size of *n* = 1528. A detailed analysis of participant attrition is described by Manz and colleagues [47]. The dropout analyses showed significant differences in socio-demographics as well as health behaviour, e.g. the response rate was significantly lower in men compared to women [47]. To partially prevent systematic bias, we controlled for the important socio-demographic factors by including the variables sex and education. In line with the scoring guidelines participants with more than one missing value in the depressive symptom inventory [48] were excluded, resulting in a final sample of 1402 young adults (mean age = 22.14 years, SD = 3.09, 46% male). Most of the participants had a moderate education level (81.9%), 11.8% had a high education level and 6.3% had a low education level, according to the CASMIN classification index [49]. A low education level is characterized by 8–9 years of schooling without formal graduation in combination with unknown, missing or completed vocational education training programmes. A moderate educational level is characterized by graduation from secondary school or advanced. A high educational level is characterized by a university degree or a degree from an university of applied science [49]. Table 1 summarizes the present sample characteristics. For the analyses, all first-order interactions were added as variables. The final dataset thus contained 190 variables.

**Table 1:** Descriptive statistics of the sample of German young adults in total and grouped by sex

						Female					Male				
Variables	*N* = 1402					*n* = 754 (54%)					*n =* 648 (46%)				
	*M*	*(SD)*	*Median*	*Min.*	*Max.*	*M*	*(SD)*	*Median*	*Min.*	*Max.*	*M*	*(SD)*	*Median*	*Min.*	*Max.*
Age	22.14	3.09	22	18	29	22.07	3.11	22	18	28	22.22	3.07	22	18	29
Depressive symptoms^a^	5.42	4.04	4.000	0	25	5.91	4.24	5	0	25	4.85	3.71	4	0	24
MVPA^b^	48.00	22.21	45.04	2.88	173.25	44.00	19.78	41.696	2.88	120.41	52.65	23.93	48.90	7.00	173.25
Media Use^c^															
PC	2.95	1.25	2.5	0.5	4.5	2.99	1.26	2.5	0.5	4.5	2.90	1.24	2.5	0.5	4.5
TV	2.96	1.21	3	0.5	4.5	3.05	1.21	3.5	0.5	4.5	2.86	1.21	2.5	0.5	4.5
Social Media	1.94	1.04	1.5	0.5	4.5	2.11	1.10	1.5	0.5	4.5	1.74	0.93	1.5	0.5	4.5
Console	1.45	1.31	0.5	0.5	4.5	0.98	0.95	0.5	0.5	4.5	2.01	1.45	1.5	0.5	4.5
Perceived Social Support^d^	86.7	15.63	94.0	9.0	100	89.86	13.38	97.00	38.00	100	83.02	17.20	88.00	9.0	100
Personality^e^															
O	5.11	1.95	5	2	10	4.90	1.97	5	2	10	5.35	1.90	5	2	10
C	7.22	1.55	7	3	10	7.53	1.44	8	3	10	6.87	1.61	7	3	10
E	6.83	1.91	7	2	10	6.96	1.90	7	2	10	6.69	1.91	7	2	10
A	5.63	1.55	6	2	10	5.55	1.50	6	2	10	5.73	1.60	6	2	10
N	5.79	1.81	6	2	10	6.25	1.77	6	2	10	5.26	1.70	5	2	10

*Note*: *MVPA* Moderate to Vigorous Physical Activity; *O* Openness; *C* Conscientiousness; *E* Extraversion; *A* Agreeableness, *N* Neuroticism;

^a^Sum score of 9 items answered on a 4-point scale from 0 (“Not at all”), 1 (“On single days”), 2 (“On more than half of the days”), 3 (“Almost every day”);

^b^Accelerometer data, averaged minutes per day over a period of one week;

^c^Self-reported average daily consumption answered on a 6-point scale from 0 (“Not at all”), 1 (“Up to 1 h”), 2 (“1 up to 2 h”), 3 (“2 up to 3 h”), to 4 (“3 up to 4 h”), 5 (“More than 4 h”);

^d^Transformed sum score ranging from 0 to 100 based on 8 items answered on a 5-point scale from 1 (“Never”) to 5 (“Always”);

^e^Mean of two items for each dimension answered on a 5-point scale from 1 (“Disagree strongly”) to 5 (“Agree strongly”)

### Measures

#### Depressive symptoms

Depressive symptoms as outcome variables were measured with the PhQ-9 scale [50]. The instrument includes nine items indicating depressive symptoms (e.g., loss of energy and interest) answered on a 4-point scale from 0 (“not at all”) to 3 (“almost every day”). A sum score was computed, and the internal consistency of the scale was Cronbach’s Alpha = 0.81.

#### Media use

To measure media use, participants indicated the average duration of their daily media consumption for the categories of social media, PC, TV and console, which were graded on the following 6-point scale: 0 (“not at all”) to 5 (“more than 4 hours”). The items were developed by Mauz and colleagues [45].

#### Physical activity

Accelerometer (GT3X+; ActiGraph LLC, Pensacola, FL, USA) data were used as an indicator of physical activity. Participants were instructed to wear the devices on their left or right hip for 7 consecutive days during the daytime. The wearing time was defined as the time the accelerometer was worn on the hip. The included participants had a wearing time of at least 8 h for a minimum of 4 days over a period of one week. The continuous accelerometer data were determined by the cut points of Troiano and colleagues [51] for adults, which define moderate to vigorous physical activity as at least 2020 counts per minute and lack of physical activity as less than 100 counts per minute (vertical axis used) referred to as sedentary behaviour. For the moderate to vigorous physical activity variable used in the current analysis, daily average values were calculated using only days with a wear time of at least 8 h. Further details of the accelerometer measurements in KiGGS Wave 2 and the device settings can be found elsewhere [52].

#### Personality

Personality as a predictor variable was measured with the 10-item version of the Big Five Inventory (BFI-10 [53];). The questionnaire consists of two items for each of the five dimensions extraversion, neuroticism, conscientiousness, agreeableness, and openness answered on a 5-point scale from 1 (“strongly disagree”) to 5 (“strongly agree”). The mean values of the two items per dimension were computed. The inter-item correlations for the extraversion, neuroticism, conscientiousness, agreeableness and openness subscales were 0.50 (*p* < .001), 0.28 (*p* < .001), 0.25 (*p* < .001), 0.10 (*p* < .001) and 0.31 (*p* < .001), respectively.

#### Perceived social support

Perceived social support was measured using 8 items that address the perceived levels of social resources and social contacts (e.g., Is there someone in your life who listens to you when you feel the need to talk?; these items were modified according to Sherbourne & Stewart, [54]. Answers were given on a 5-point rating scale from 1 (“never”) to 5 (“always”) and were summarized and transformed to a standardized scale with a minimum of 0 and a maximum of 100 [54]. The internal consistency of the scale was Cronbach’s Alpha = 0.90.

#### Covariates

Accelerometer wear time and correlates of depressive symptoms were included as control variables in the present study. This includes the socioeconomic status of the parents [55, 56], the participant’s education level [57], age [58], sex [59] and personal resources. We operationalized personal resources with 5 self-developed items (e.g., meaningful life) rated on a 4-point rating scale from 1 (“not true”) to 4 (“exactly right”). For example, previous research had identified a meaningful life as a preventive factor for suicide [60] and depression [61]. The internal consistency of the scale was Cronbach’s Alpha = 0.80.

### Data analyses

First, missing item values (education level had the greatest number of missing values (*n* = 50) and perceived social support had the fewest (*n* = 1)) were replaced at the score level by predictive mean matching (PMM) in R (package ‘mice’, version 3.8.0, Stef van Buuren). Within this process, five imputations per missing observation were generated and thereafter pooled for analyses. The procedure of multiple imputation made it possible to obtain the largest possible sample, as the analyses do not allow any missing values in the data set. The goal was to find a fitting and stable regression model to statistically predict depressive symptoms, considering all possible variables and first-order interactions. We performed stepwise, lasso, ridge and elastic net regression analyses to predict depressive symptoms by media use, physical activity, personality and perceived social support. Moreover, we included the participants’ education level, age, sex, accelerometer wear time, personal resources and parental socioeconomic status as control variables in the models. Since the study’s objective focuses on moderation effects, all possible first-order interactions were included. In addition, wear time was included as a main effect control variable. The continuous predictors were entered as z-standardized scores.

The advantage of this statistical approach is the utilization of a comparison of statistical models that offer an automated selection of significant variables in consideration of the entire data set (ridge and elastic net regression; 63). The reason behind this is that a valid result should be significant under the consideration of all possible alternative or parallel associations. By including all possible variables and first-order interactions, the analyses control for all variables and interactions simultaneously. Variables with low levels of contribution to the explanation of variance in depressive symptoms are statistically excluded. However, the inclusion of multiple variables overstresses a multiple linear regression model because of multicollinearity and overfitting but can be dealt with by lasso, ridge and elastic net regression and ten-fold cross-validation [62, 63]. The root mean square error (RMSE), mean absolute error (MAE) and R-squared served as the decision criteria for the best model fit. Additionally, we performed simple slope analyses for significant interactions (Table 2).

**Table 2:** Simple slope analyses for significant interactions resulting from elastic net regression on depressive symptoms

*Predictor*	*Moderator (1 SD below, 1 SD above and at the mean level)*		*Estimate*	*Std. Error*	*p*
MVPA	Extraversion ^c^	4.92	−.017	.01	.007**
		6.83	−.011	.00	.018*
		8.74	−.005	.01	.467
	Neuroticism ^c^	3.99	−.002	.01	.764
		5.79	−.008	.00	.074
		7.60	−.014	.01	.031 *
	Conscientiousness ^c^	5.67	−.010	.01	.186
		7.22	−.010	.00	.044 *
		8.77	−.010	.01	.116
Sedentary behaviour	Extraversion ^c^	4.92	.002	.00	.319
		6.83	−.001	.00	.406
		8.74	−.003	.00	.036 *
PC ^a^	Agreeableness ^c^	4.09	.319	.12	.007 **
		5.63	.503	.08	<.001 ***
		7.18	.687	.12	<.001 ***
	Openness ^c^	3.16	.624	.12	<.001 ***
		5.11	.530	.09	<.001 ***
		7.06	.435	.12	<.001 ***
	Perceived Social Support ^b^	71.06	.633	.11	<.001 ***
		86.70	.518	.08	<.001 ***
		102.33	.404	.12	.001 ***
TV ^a^	Conscientiousness ^c^	5.67	.532	.12	<.001 ***
		7.22	.317	.09	<.001 ***
		8.77	.102	.13	.419
	Agreeableness ^c^	4.09	.300	.12	.014 *
		5.63	.419	.09	<.001 ***
		7.18	.537	.12	<.001 ***
Social Media ^a^	Extraversion ^c^	4.92	.367	.14	.011 *
		6.83	.475	.10	<.001 ***
		8.74	.583	.14	<.001 ***

*Note*: *MVPA* Moderate to Vigorous Physical Activity;

^a^Self-reported average daily consumption answered on a 6-point scale from 0 (“Not at all”), 1 (“Up to 1 h”), 2 (“1 up to 2 h”), 3 (“2 up to 3 h”), to 4 (“3 up to 4 h”), 5 (“More than 4 h”);

^b^Transformed sum score ranging from 0 to 100 based on 8 items answered on a 5-point scale from 1 (“Never”) to 5 (“Always”);

^c^Mean of two items for each dimension answered on a 5-point scale from 1 (“Disagree strongly”) to 5 (“Agree strongly”)

## Results

### Model comparison

Table 3 shows the model fit indices. The elastic net regression showed the best fit (α = 0.111 and λ = 0.25) and had the smallest RMSE (Table 3).

**Table 3:** Model fit indices resulting from stepwise, Ridge, Lasso and Elastic Net Regression

*MAE*	*Min.*	*1st Qu.*	*Median*	*M*	*3rd Qu.*	*Max.*
Linear Model	.592	.664	.703	.702	.752	.818
Ridge	.548	.624	.652	.653	.688	.748
Lasso	.604	.674	.700	.703	.730	.799
**Elastic Net**	**.542**	**.601**	**.629**	**.630**	**.667**	**.724**
*RMSE*						
Linear Model	.757	.857	.936	.928	.988	1.102
Ridge	.670	.783	.865	.855	.908	1.012
Lasso	.710	.854	.913	.912	.977	1.150
**Elastic Net**	**.645**	**.780**	**.829**	**.828**	**.881**	**0.996**
*R*^*2*^						
Linear Model	.068	.144	.209	.220	.286	0.512
Ridge	.081	.204	.261	.271	.335	0.488
Lasso	.057	.194	.228	.241	.294	0.408
**Elastic Net**	**.110**	**.261**	**.310**	**.334**	**.404**	**0.558**

*Note:* The best model fit resulting from Elastic Net Regression is highlighted in boldface

**Fig. 1:**
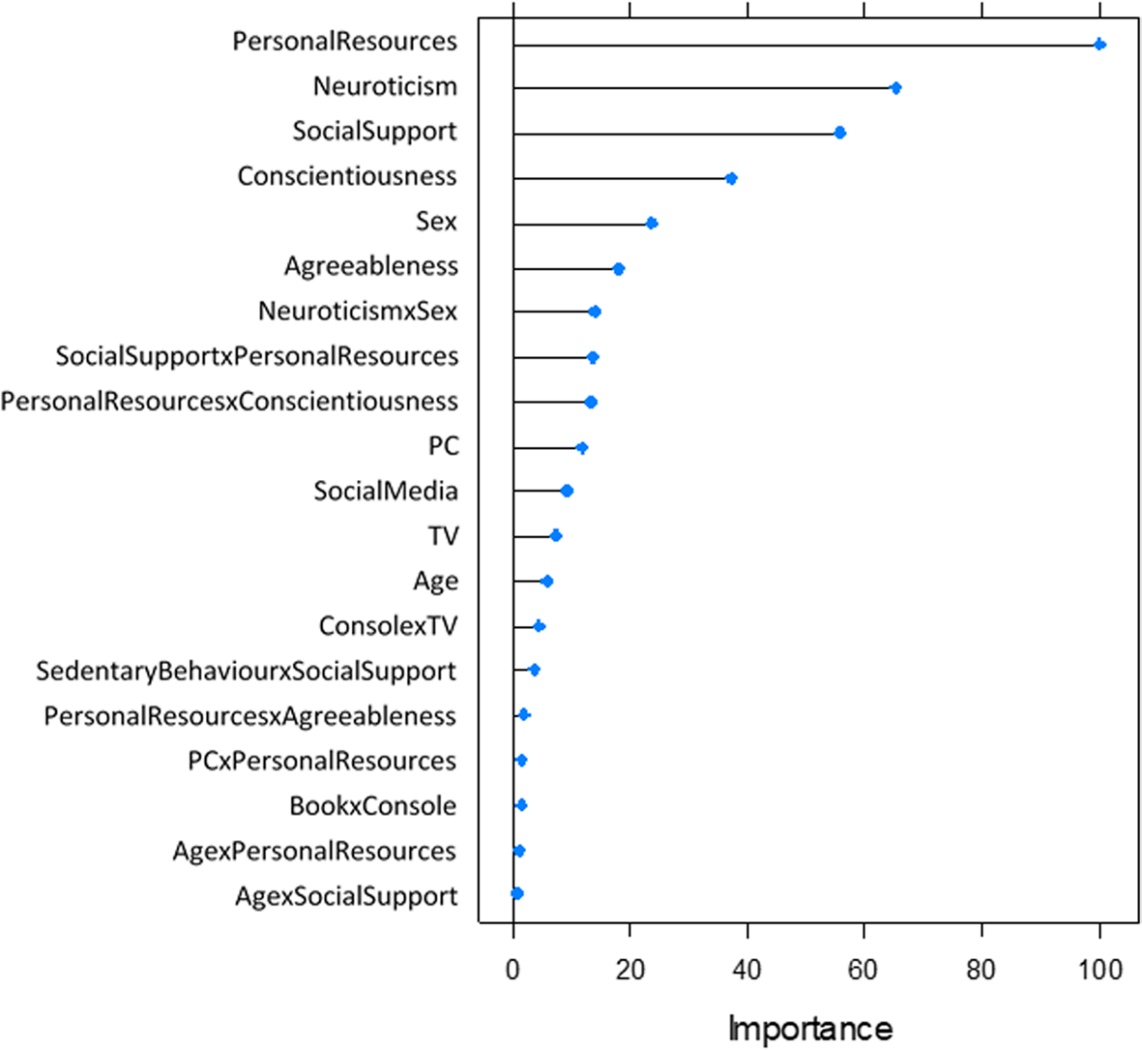
Top 20 variable importance as indicator of the contribution to reduce the estimation error in the prediction of depressive symptoms

Figure 1 shows the variable importance including the 20 best variables and interactions in descending order extracted by the elastic net regression model. Variable importance expresses the changes in the generalized cross-validation for each predictor and calculates the reduction in the statistic when each predictor’s feature is added to the model, which occurs relative to the maximum [62]. A higher variable importance indicates a higher contribution to reduce the estimation error and to predict depressive symptoms. The model was able to reduce the dataset from 190 to 66 significant variables and explained 33.4% of the variance in depressive symptoms (Table 3). The importance levels of all remaining variables are shown Fig. A1 in the Additional file 1.

### Main effects on depressive symptoms

Media use (social media, PC and TV) was positively associated with depressive symptoms whereas physical activity was not (Fig. 1). Neuroticism and agreeableness were positively correlated with depressive symptoms. Conscientiousness, perceived social support and age were negatively associated with depressive symptoms.

### Personality and perceived social support as moderators of the relationship between media use and depressive symptoms

**Fig. 2:**
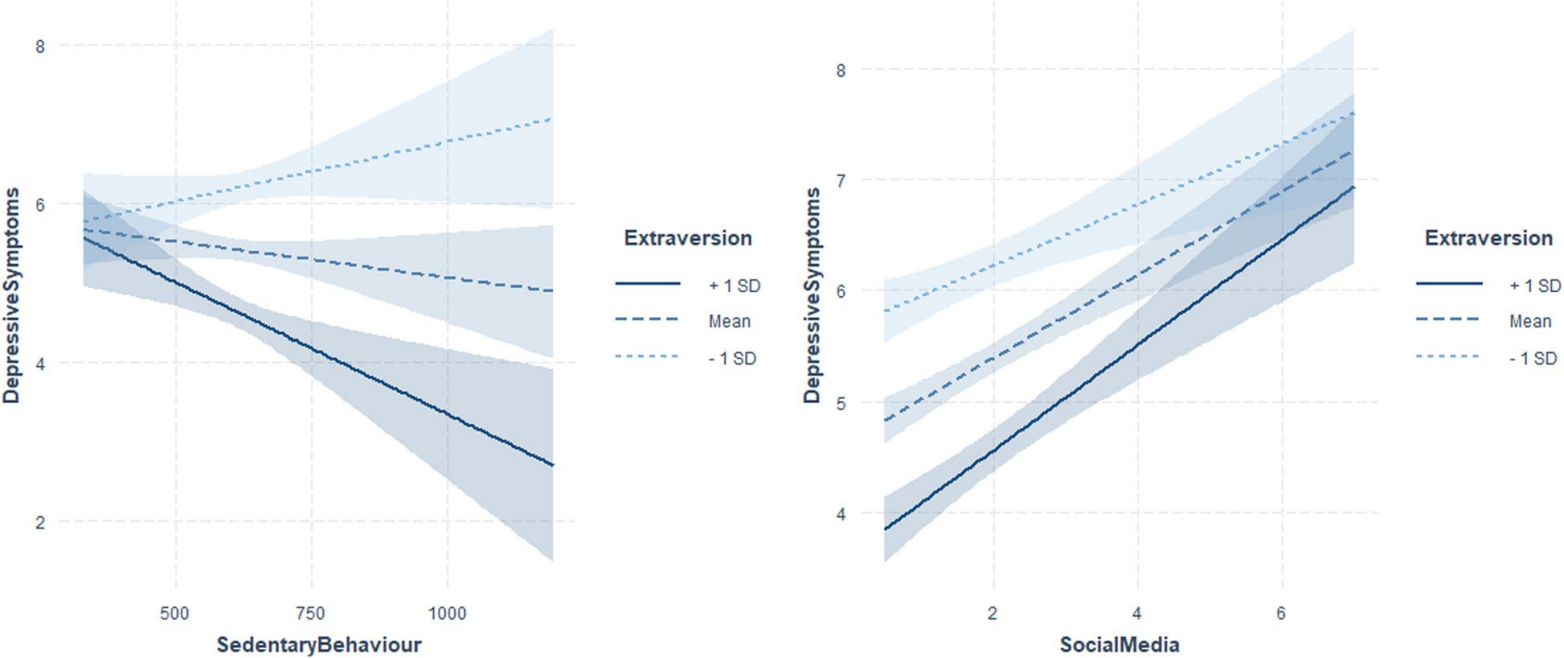
Interaction plots showing simple slopes of health-related behaviours with the moderator extraversion predicting depressive symptoms (min = 0, max = 25) for 1 SD below (5.06), 1 SD above (8.86) and at the mean level of extraversion (*M* = 6.96). Coloured shading represents 95% CIs

**Fig. 3:**
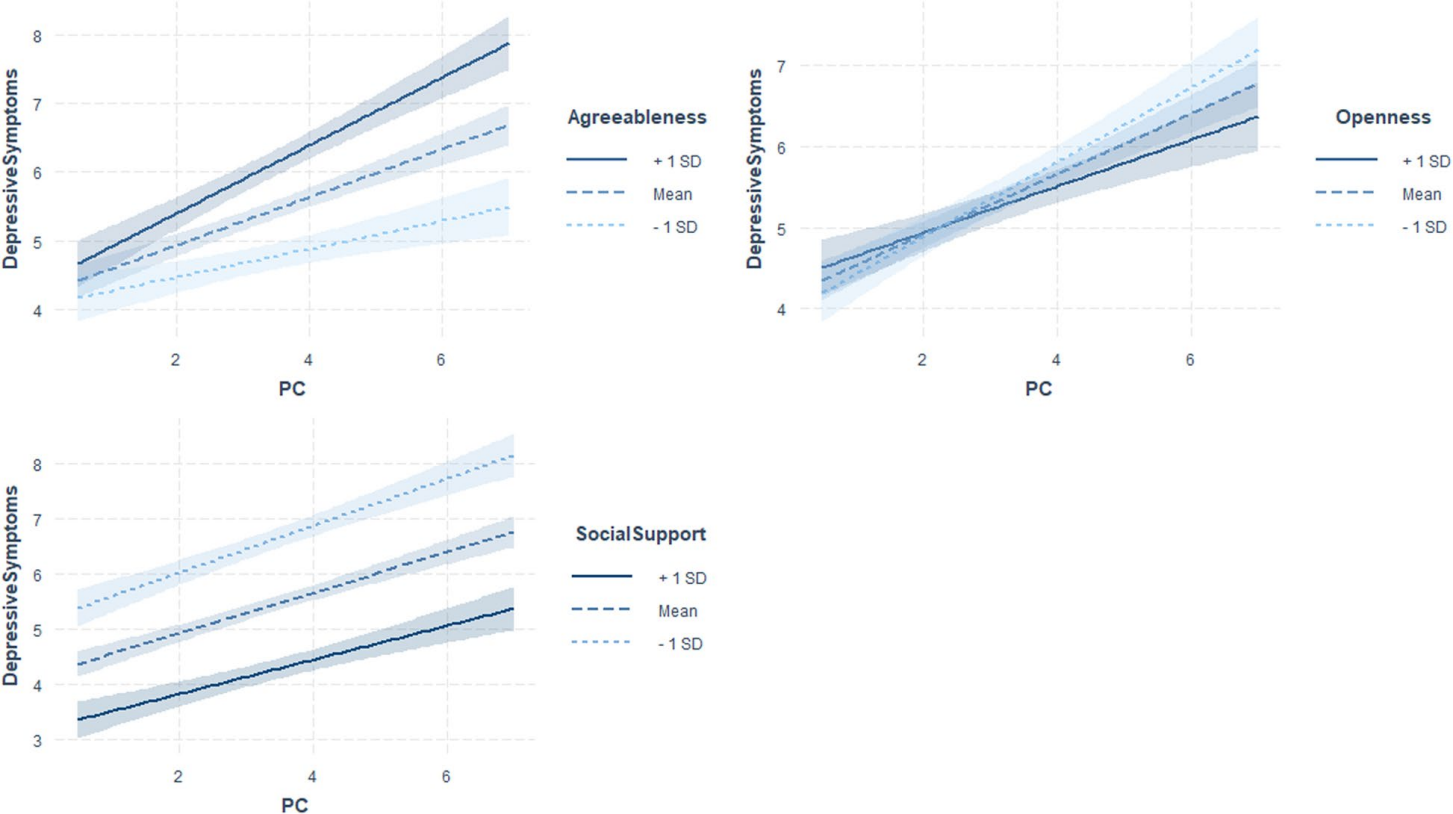
Interaction plots showing simple slopes of PC use predicting depressive symptoms (min = 0, max = 25) for 1 SD below (4.04), 1 SD above (7.05) and at the mean level of agreeableness (*M* = 5.55); for 1 SD below (2.93), 1 SD above (2.93) and at the mean level of openness (*M* = 4.90); for 1 SD below (76.48), 1 SD above (103.24) and at the mean level of social support (*M* = 89.86). Coloured shading represents 95% CIs

**Fig. 4:**
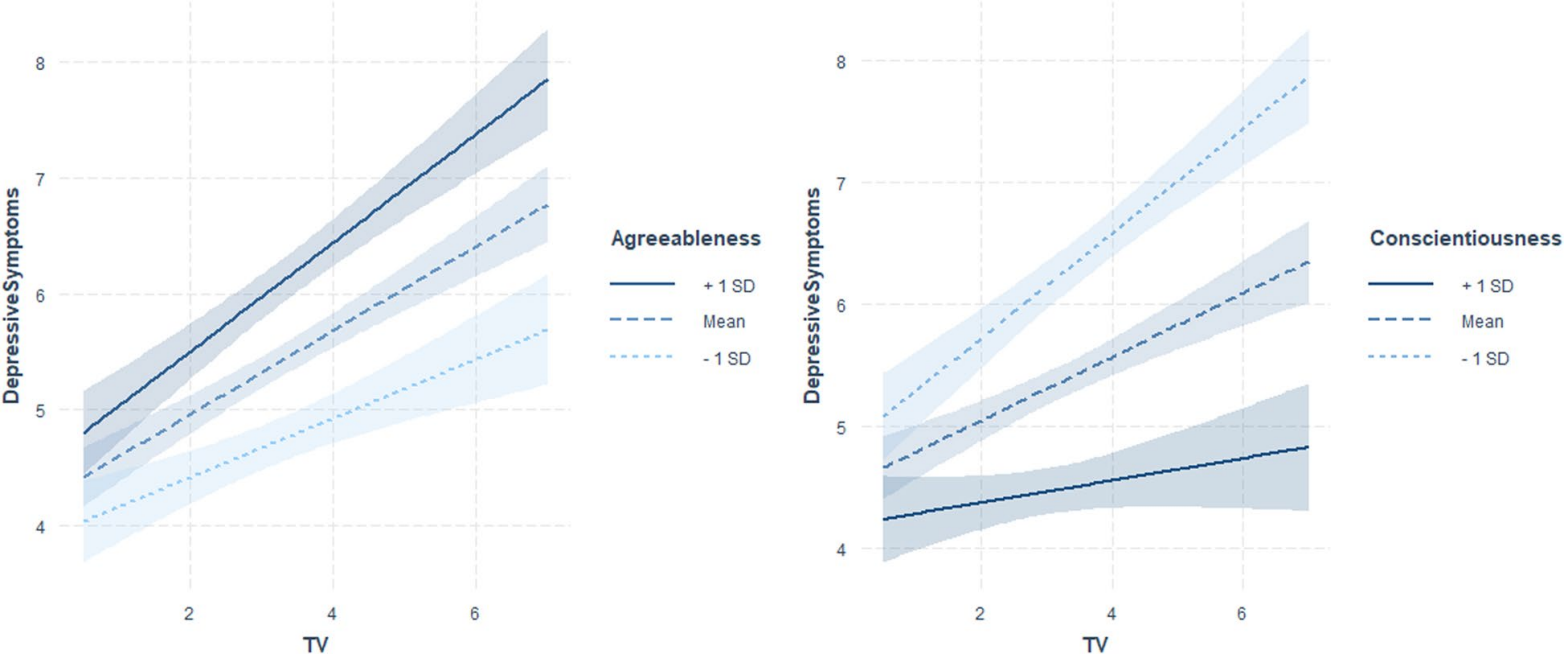
Interaction plots showing simple slopes of TV use predicting depressive symptoms (min = 0, max = 25) for 1 SD below (4.04), 1 SD above (7.05) and at the mean level of agreeableness (*M* = 5.55); for 1 SD below (6.09), 1 SD above (8.97) and at the mean level of conscientiousness (*M* = 7.53) and for 1 SD below (0.03), 1 SD above (1.93) and at the mean level of playing console (*M* = 0.98). Coloured shading represents 95% CIs

Personality traits moderated the positive association between media use and depressive symptoms (Figs. 2, 3 and 4).

Extraverts showed a stronger positive association of social media use with depressive symptoms (Fig. 2, Table 2). However, individuals with high levels of perceived social support showed a weaker positive association of PC use with depressive symptoms (Fig. 3, Table 2). The opposite pattern was found for individuals with low levels of openness. Persons with low levels of conscientiousness showed a stronger positive association of TV use with depressive symptoms, and persons with high levels of agreeableness showed a stronger positive association of TV use and PC use with depressive symptoms (Figs. 3 and 4, Table 2). Significant interactions of the predictors with control variables are visualized in Figs. A2 to A4 in the Additional file 1.

### Personality and perceived social support as moderators of the relationship between physical activity and depressive symptoms

**Fig. 5:**
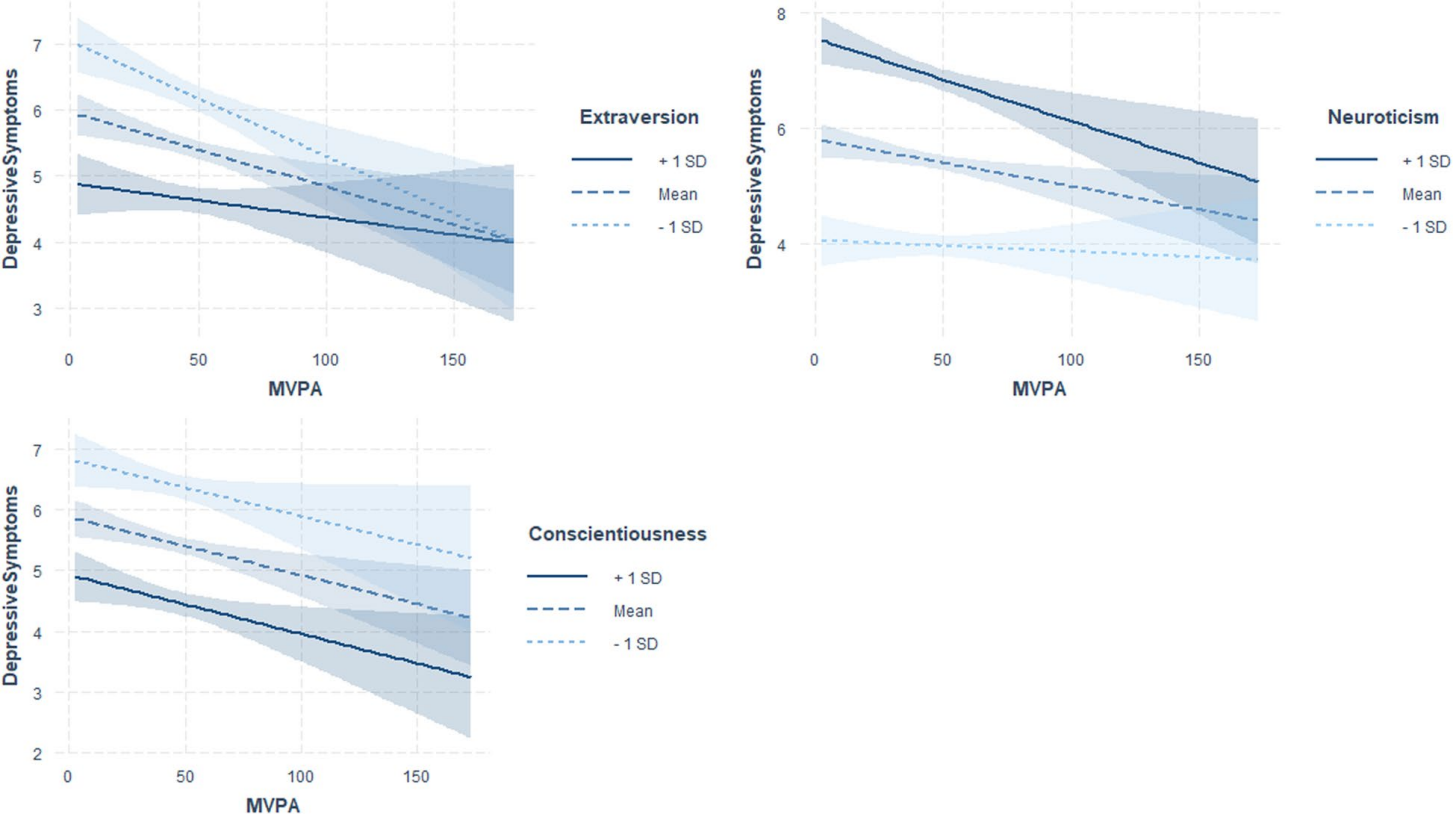
Interaction plots showing simple slopes of moderate to vigorous physical activity (MVPA) predicting depressive symptoms (min = 0, max = 25) for 1 SD below (5.06), 1 SD above (8.86) and at the mean level of extraversion (*M* = 6.96); for 1 SD below (4.48), 1 SD above (8.02) and at the mean level of neuroticism (*M* = 6.25); for 1 SD below (6.09), 1 SD above (8.97) and at the mean level of conscientiousness (*M* = 7.53). Coloured shading represents 95% CIs

Personality traits moderated the association between moderate to vigorous physical activity and depressive symptoms (Fig. 5, Table 2). Individuals with low to average levels of extraversion and high levels of neuroticism showed a stronger negative association between moderate to vigorous physical activity and depressive symptoms. Additionally, extraverts showed a weaker positive association between sedentary behaviour and depressive symptoms. Perceived social support did not moderate the associations between physical activity and depressive symptoms.

Further exploratory results on interactions that were not within the present research scope can be obtained from the Supplementary Materials Table A1 and Figs. A2 to A4.

## Discussion

In the present study, we investigated the role of personality traits and perceived social support as moderators of the relationships of physical activity and media use with depressive symptoms in a German population sample of young adults. We hypothesized that personality traits and perceived social support moderate (H1) the association between media use and depressive symptoms and (H2) the association between physical activity and depressive symptoms. By comparing different regression models, we selected the best model fit (elastic net) containing a selection of the most relevant predictors of depressive symptoms and their interactions. The results suggest that associations of media use and physical activity with depressive symptoms were moderated by personality and perceived social support, as discussed below.

### The role of personality traits and perceived social support in associations between media use and depressive symptoms

In accordance with H1, our results show that personality traits and perceived social support moderated the association between media use and depressive symptoms.

Unexpectedly, positive associations between social media use and depressive symptoms appeared to be stronger amongst extraverted participants than amongst introverted participants. Considering the frequently reported positive associations between extraversion and mental health [64], we would have expected the opposite pattern. The present data do not allow for a substantive explanation, however, for future studies the selective exposure theory (SET [23];) represents a good starting point. SET proposes that extraverts tend to disclose their plain self [23] and thus may reveal more personal details compared with more restrictive user types and thereby provide a greater scope for negative and harmful feedback. For example, oversharing (i.e., the unrestrictive behaviour of sharing information and pictures of one’s life) has been reported as a potential risk of cyberbullying by social media users [65]. In accordance, other research has found that sharing attention-seeking posts leads to negative experiences for sharing individuals [65]. High levels of sharing behaviour may result in more frequent social comparisons in an atmosphere of optimized selves, e.g., on Facebook [32], that may in turn negatively affect extraverts. Another possible explanation might be related to the fact that individuals with depressive symptoms tend to use social media more often than their counterparts [65], as do extraverts compared to introverts [11, 31]. It can be assumed that while in a depressive state, an extravert might show more frequent social media use than an introverted person due to generally engaging in more frequent social contacts in offline relationships. Consequently, the results could indicate that extraverts tend to use social media more often, particularly when experiencing depressive symptoms. In favour of this assumption, Määttänen and colleagues [66] more generally argued that personality-like affect states may be a better predictor of behaviour such as physical activity than trait measures. They emphasise the need for more detailed research based on a situational approach to help further understand causal relationships. However, these ad-hoc assumptions require further (longitudinal) investigations, including more detailed information on social media behaviour and content.

Low levels of conscientiousness and high levels of agreeableness showed stronger positive associations of PC use with depressive symptoms as well as of watching TV with depressive symptoms. These findings are in line with previous results regarding the health risk behaviours of individuals with low levels of conscientiousness. A previous study reported that low levels of conscientiousness were related to risk behaviour; conversely, highly conscientious individuals seemed to be more motivated to meet health-related norms and recommendations [67]. Furthermore, conscientiousness, which is defined as an organized and disciplined personality, might have a compensatory function in terms of well-being, thereby enhancing the probability that everyday duties are met even while spending long hours in front of a TV screen. Consequently, low levels of conscientiousness in combination with excessive TV screen time might be associated with unattended daily responsibilities, resulting in a growing number of life problems. This argument matches a previous discussion that considered low levels of conscientiousness to be closely related to negative health consequences [68]. Similarly, the combination of low levels of conscientiousness and risk behaviour, such as extensive media use, seems to be associated with the risk of negative mental health consequences.

A possible explanation for the increased associations of media use with depressive symptoms among individuals with high levels of agreeableness could be that PC or TV use is in contradiction to their need for direct interpersonal contact [69]. In this case, TV use could be seen as a placeholder for a lack of social interaction, the latter being a particularly strong need for people with high agreeableness scores.

Furthermore, persons with low levels of openness showed a stronger positive association of PC use with depressive symptoms. In general, low levels of openness were shown to be related to social anxiety [70], and in the context of media use, such low levels may imply social withdrawal, which in turn represents a risk factor for depressive symptoms [71]. This argument is supported by results showing that loneliness is negatively related to openness [72]. However, this theory-based attempt to classify our results in terms of content requires further evidence.

Individuals with high levels of perceived social support showed a weaker positive association of PC use with depressive symptoms than did those with low levels of perceiving social support, as expected in H1. The restricting factor is that we do not have any information about the media content. Based on the idea that individuals have a fulfilling social life offline and engage with social media via their PC to strengthen social relationships [65, 73], the results suggest positive associations with mental health. The crucial fact that must be considered is that social media use should not function as compensation for unmet real-life needs, such as a lack of social relationships or perceived social support [65, 73]. Therefore, future research should take the media content and motivations of use into account to better understand the identified associations. The same applies for a better understanding of the role of neuroticism in associations between media use and depressive symptoms. In contrast to our expectations, neuroticism did not moderate the association between media use and depressive symptoms. Based on theoretical assumptions, we would have expected a negative effect resulting from differing use motives (i.e., emotional self-disclosure [23];, online behaviour (i.e., presenting an ideal self-image [23];) and processes *during* media use (i.e., social comparison [32];).

### The role of personality traits and perceived social support in associations between physical activity and depressive symptoms

In line with H2, personality traits and perceived social support also moderated the relationship between physical activity and depressive symptoms.

We noted that extraverts showed a weaker negative association between moderate to vigorous physical activity and depressive symptoms as compared to introverts. Emotionally labile individuals showed a stronger negative association between moderate to vigorous physical activity and depressive symptoms than emotionally stable ones.

That the negative association between moderate to vigorous physical activity and depressive symptoms was weaker in extraverted young adults than in introverts, supported our hypothesis. Explanations can be derived from the biological mechanisms of physical activity summarized by Kandola and colleagues in their review [38]. Both the inflammation aspect and the neuroendocrinological aspect represent a biological imbalance that can affect depressive symptoms as a result of chronic stress. However, during a depressive episode, the level of extraversion seems to be temporarily decreased [74, 75]. Keeping this in mind, the results might indicate that high levels of extraversion and severe depressive symptoms are mutually exclusive and that high levels of extraversion can be seen as an indication of only mild to moderate depressive symptoms. Additionally, lower levels of depressive symptoms have been related to smaller biological imbalances [38]. Hence, physical activity, as a general potential compensatory factor, cannot reach its full potential in highly extraverted people due to an already more balanced biological state. In order to substantiate these theoretical considerations and to understand the contextual relationships, it is necessary to collect different physiological parameters. This corresponds to Määttänen and colleagues [76] who suspect situational changes in physiological parameters underlying depressive symptoms. Another idea is that the negative association of physical activity with depressive symptoms is also influenced by the social interactions [38] that occur during physical activities reducing social isolation. For highly extraverted individuals, this mechanism might be less effective due to an already fulfilled social life [77].

Moreover, our results suggest that emotionally labile individuals have a stronger negative association between moderate to vigorous physical activity and depressive symptoms. One possible explanation refers to a preconditioned biopsychological imbalance of emotionally labile persons. For example, neuroticism has been related to a disproportionate negative affectivity [30] and longer lasting recovery in response to stress (e.g., as indicated by cortisol release [78];). Thus, the collection of physiological parameters such as heart rate variability in follow-up studies could be beneficial for the investigation of individual depressive symptoms, as suggested by Määttänen and colleagues [76].

Low levels of extraversion showed a stronger positive association between sedentary behaviour and depressive symptoms. This finding is difficult to interpret since we have no information about the context of sedentary behaviour. A few results point towards toward the possibility that sedentary behaviour can be interpreted as a consequence of social withdrawal [79], which is listed as a risk factor for depression [71]. This would offer an explanation why introverts showed a stronger positive association between sedentary behaviour and depressive symptoms.

### Strengths and limitations

This study is characterised by an extensive statistical approach that takes a broad variety of relevant variables into account. The statistical regularization procedure of the elastic net regression model led to a reduced bias-variance trade-off. Both aspects increased the validity of the results. Additionally, the validity profits from the broad population sample of young adults, representing a high-risk group for depressive symptoms. The results furthermore benefit from the use of accelerometer data as an objective indicator for physical activity and reduced distortion effects due to self-report measures.

However, several limitations must be taken into account when interpreting the results of the present study. First, the sample does not meet the criteria of representativeness due to longitudinal dropout of the cohort participants, selection bias, and further attrition due to non-response, technical problems or non-compliance regarding accelerometry. Thus, results cannot be generalized to the young adult population living in Germany. Second, the data quality of media use based on self-reports has been considered controversial [31] and represents only an indicator of screen time. Third, information on the motivation for media use and media content was not available. The measurement of personality with only two items per dimension is only a broad indicator and should be measured with more comprehensive inventories in future studies. As a result, the measurement of the effects of the personality variables may be severely underestimated, which would explain the contradiction with existing literature. Finally, we cannot clarify predictive direction because of the cross-sectional design of the dataset. Future research should establish a longitudinal design to further contribute to the understanding of differential effects of personality differences on the association of health risk behaviours with depressive symptoms.

## Conclusion

The present results suggest that personality traits and perceived social support play a role in understanding individual differences in the associations between health-related behaviours such as media use and physical activity and depressive symptoms.

Furthermore, the results show that objective physical activity and media use data without further information on relevant individual characteristics do not allow general classification of their functioning as protective factors or risk behaviours. In particular, the present findings suggest that health protective effects of health-related behaviour may vary as a function of personality and perceived social support. Further replication and extended evidence on relevant individual characteristics involved in the moderation of depressive symptoms and health-related behaviour can help establish tailored intervention.

## Supplementary Information

**Additional file 1: Fig. A1.** Variable importance of all the included variables as indicator of the contribution to reduce the estimation error in the prediction of depressive symptoms. **Fig. A2.** Interaction plots showing simple slopes of health risk behaviours predicting depressive symptoms (min=0, max=25) for 1 SD below (8.45), 1 SD above (15.79) and at the mean level of socioeconomic status (M=12.12); for 1 SD below (3.63), 1 SD above (4.48) and at the mean level of education (M=4.05); for 1 SD below (19.04), 1 SD above (25.23) and at the mean level of age (M=22.14); for 1 SD below (55.59), 1 SD above (83.89) and at the mean level of personal resources (M=69.74). Coloured shading represent 95% CIs. **Fig. A3.** Interaction plots showing simple slopes of health risk behaviours predicting depressive symptoms (min=0, max=25) for 1 SD below (19.04), 1 SD above (25.23) and at the mean level of age (M=22.14); for sex (46 % male); for 1 SD below (55.59), 1 SD above (83.89) and at the mean level of personal resources (M=69.74); for 1 SD below (1.75), 1 SD above (4.17) and at the mean level of TV (M=2.96). Coloured shading represent 95% CIs. **Fig. A4.** Interaction plots showing simple slopes of health risk behaviours predicting depressive symptoms (min=0, max=25) for 1 SD below (25.79), 1 SD above (70.21) and at the mean level of MVPA (M=48.00); for sex (46 % male). Coloured shading represent 95% CIs. **Table A1.** Simple slope analyses for significant interactions resulting from elastic net regression on depressive symptoms.

## Data Availability

The dataset created and analysed in the current study is not publicly available because the consent of the study participants did not cover the publication of the data. However, the data are available on request from the corresponding author.
