# Supplementary material for: The role of personality traits and social support in relations of health-related behaviours and depressive symptoms
Source: BMC Psychiatry. 2022 Jan 22;22:52. doi: 10.1186/s12888-022-03693-w (PMC8784003; doi:10.1186/s12888-022-03693-w)
Supplement: Supplementary file 1 — Additional file 1: Fig. A1. Variable importance of all the included variables as indicator of the contribution to reduce the estimation error in the prediction of depressive symptoms. Fig. A2. Interaction plots showing simple slopes of health risk behaviours predicting depressive symptoms (min=0, max=25) for 1 SD below (8.45), 1 SD above (15.79) and at the mean level of socioeconomic status (M=12.12); for 1 SD below (3.63), 1 SD above (4.48) and at the mean level of education (M=4.05); for 1 SD below (19.04), 1 SD above (25.23) and at the mean level of age (M=22.14); for 1 SD below (55.59), 1 SD above (83.89) and at the mean level of personal resources (M=69.74). Coloured shading represent 95% CIs. Fig. A3. Interaction plots showing simple slopes of health risk behaviours predicting depressive symptoms (min=0, max=25) for 1 SD below (19.04), 1 SD above (25.23) and at the mean level of age (M=22.14); for sex (46 % male); for 1 SD below (55.59), 1 SD above (83.89) and at the mean level of personal resources (M=69.74); for 1 SD below (1.75), 1 SD above (4.17) and at the mean level of TV (M=2.96). Coloured shading represent 95% CIs. Fig. A4. Interaction plots showing simple slopes of health risk behaviours predicting depressive symptoms (min=0, max=25) for 1 SD below (25.79), 1 SD above (70.21) and at the mean level of MVPA (M=48.00); for sex (46 % male). Coloured shading represent 95% CIs. Table A1. Simple slope analyses for significant interactions resulting from elastic net regression on depressive symptoms. [file 12888_2022_3693_MOESM1_ESM.docx]

**Appendix**

PersonalResources

Neuroticism

SocialSupport

Conscientiousness

Sex

Agreeableness

NeuroticismxSex

SocialSupportxPersonalResources

PersonalResourcesxConscientiousness

PC

SocialMedia

TV

Age

ConsolexTV

SedentaryBehaviourxSocialSupport

PersonalResourcesxAgreeableness

PCxPersonalResources

BookxConsole

AgexPersonalResources

AgexSocialSupport

SocialSupportxAgreeableness

ExtraversionxMVPA

TVxPersonalResources

NeuroticismxEducation

PCxAgreeableness

TVxAge

PersonalResourcesxSex

AgexSocioEconomicStatus

PersonalResourcesxNeuroticism

PCxSocialSupport

ConsolexSex

AgexConscientiousness

LightPhysicalActivityxPC

SexxEducation

LightPhysicalActivityxSocialMedia

PCxEducation

ConsolexSocialSupport

BookxSocialSupport

PCxAge

ConscientiousnessxEducation

SocialSupportxSex

AgreeablenessxSocioEconomicStatus

ConscientiousnessxMVPA

SedentaryBehaviourxExtraversion

Education

ExtraversionxAgreeableness

PCxSocioEconomicStatus

OpennessxAgreeableness

ExtraversionxEducation

SedentaryBehaviourxSocioEconomicStatus

SocialMediaxMVPA

SocioEconomicStatus

TVxAgreeableness

LightPhysicalActivityxConsole

AgexNeuroticism

NeuroticismxMVPA

ExtraversionxConscientiousness

PCxOpenness

TVxSex

ConscientiousnessxSex

SocialSupportxOpenness

BookxExtraversion

SocialMediaxExtraversion

TVxConscientiousness

AgexOpenness

OpennessxConscientiousness

**Fig. A1**. Variable importance of all the included variables as indicator of the contribution to reduce the estimation error in the prediction of depressive symptoms.


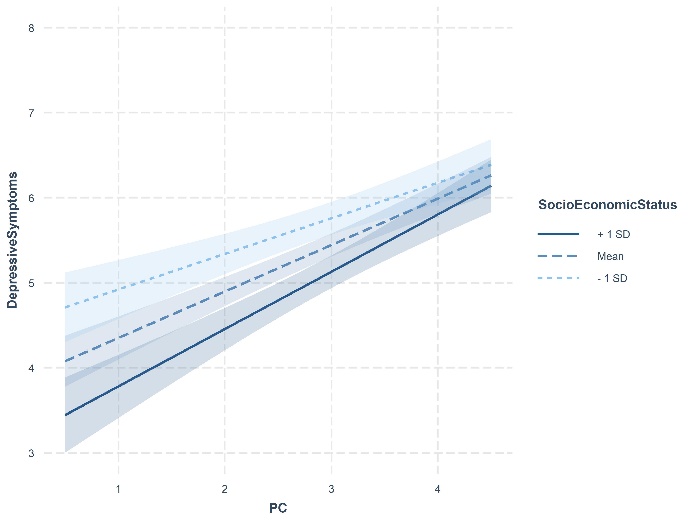

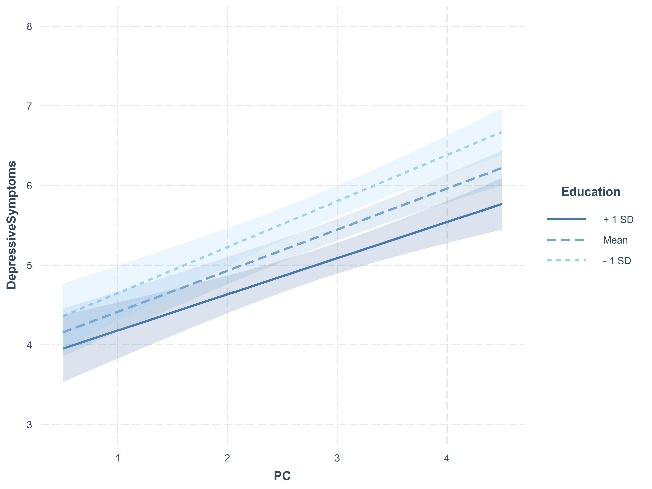


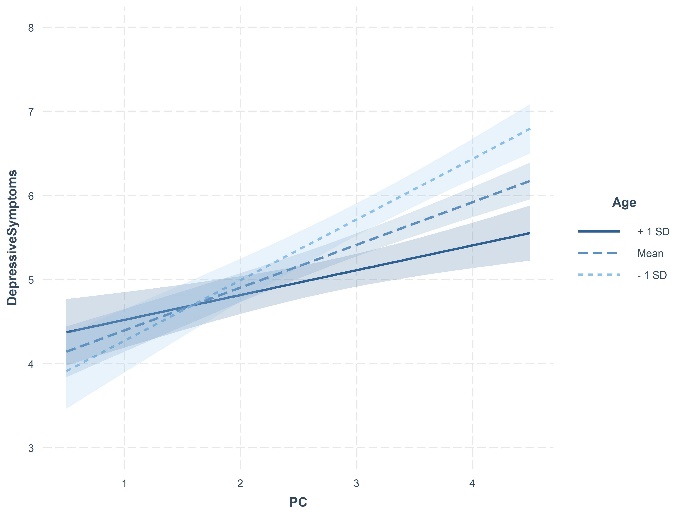

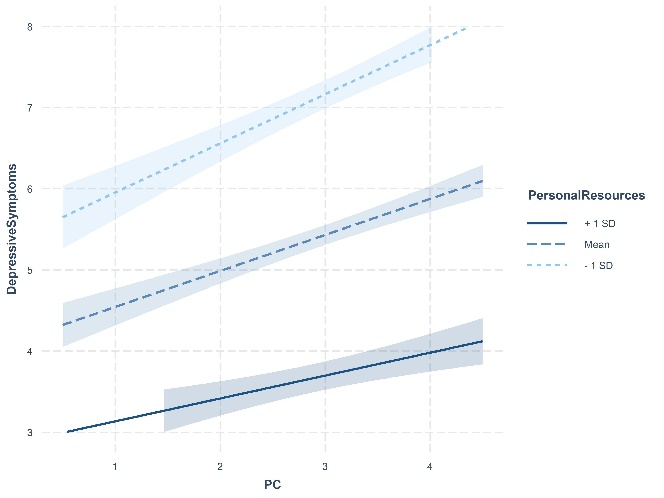


**Fig. A2**. Interaction plots showing simple slopes of health risk behaviours predicting depressive symptoms (min=0, max=25) for 1 SD below (8.45), 1 SD above (15.79) and at the mean level of socioeconomic status (*M*=12.12); for 1 SD below (3.63), 1 SD above (4.48) and at the mean level of education (*M*=4.05); for 1 SD below (19.04), 1 SD above (25.23) and at the mean level of age (*M*=22.14); for 1 SD below (55.59), 1 SD above (83.89) and at the mean level of personal resources (*M*=69.74). Coloured shading represent 95% CIs.


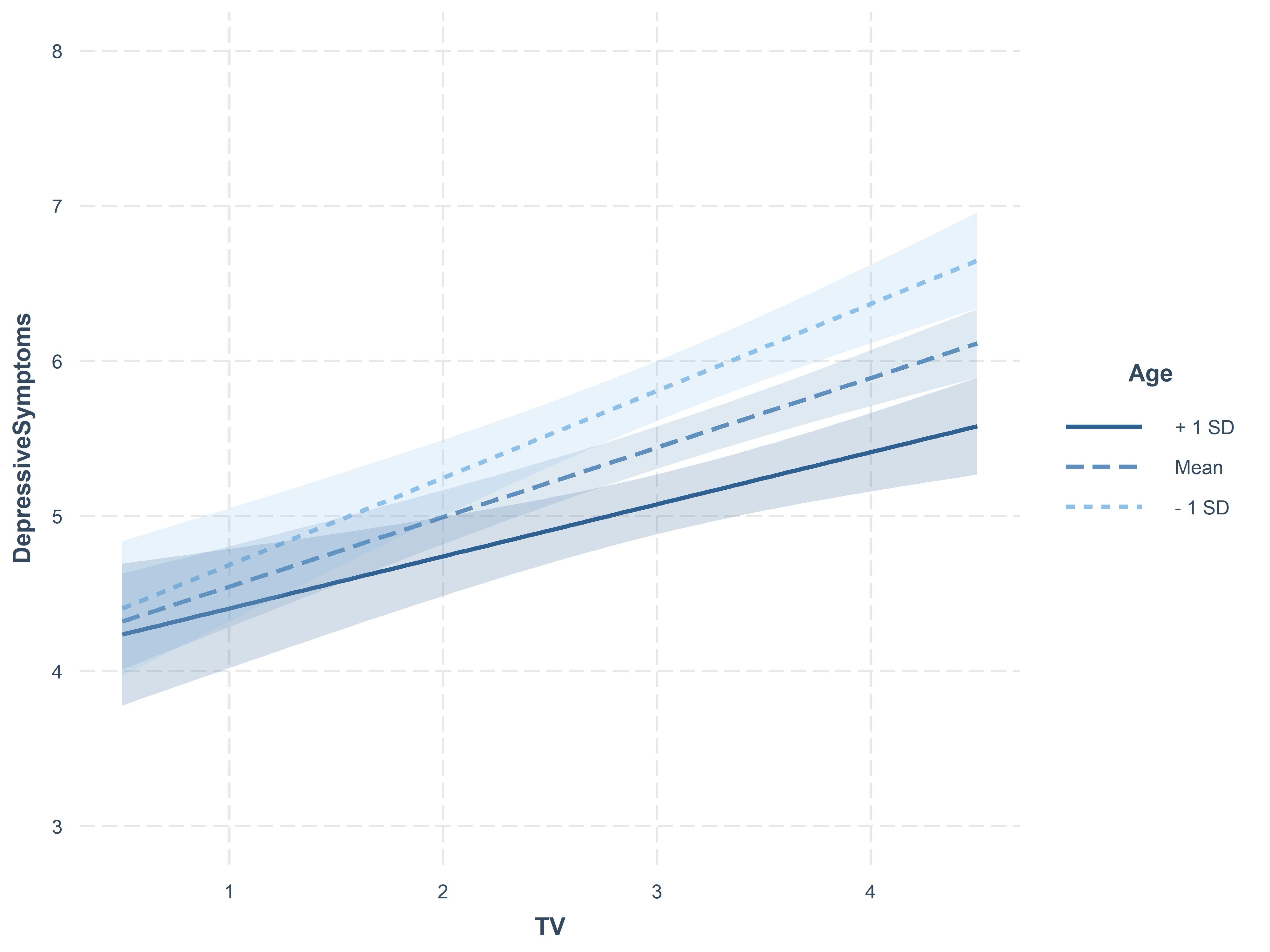

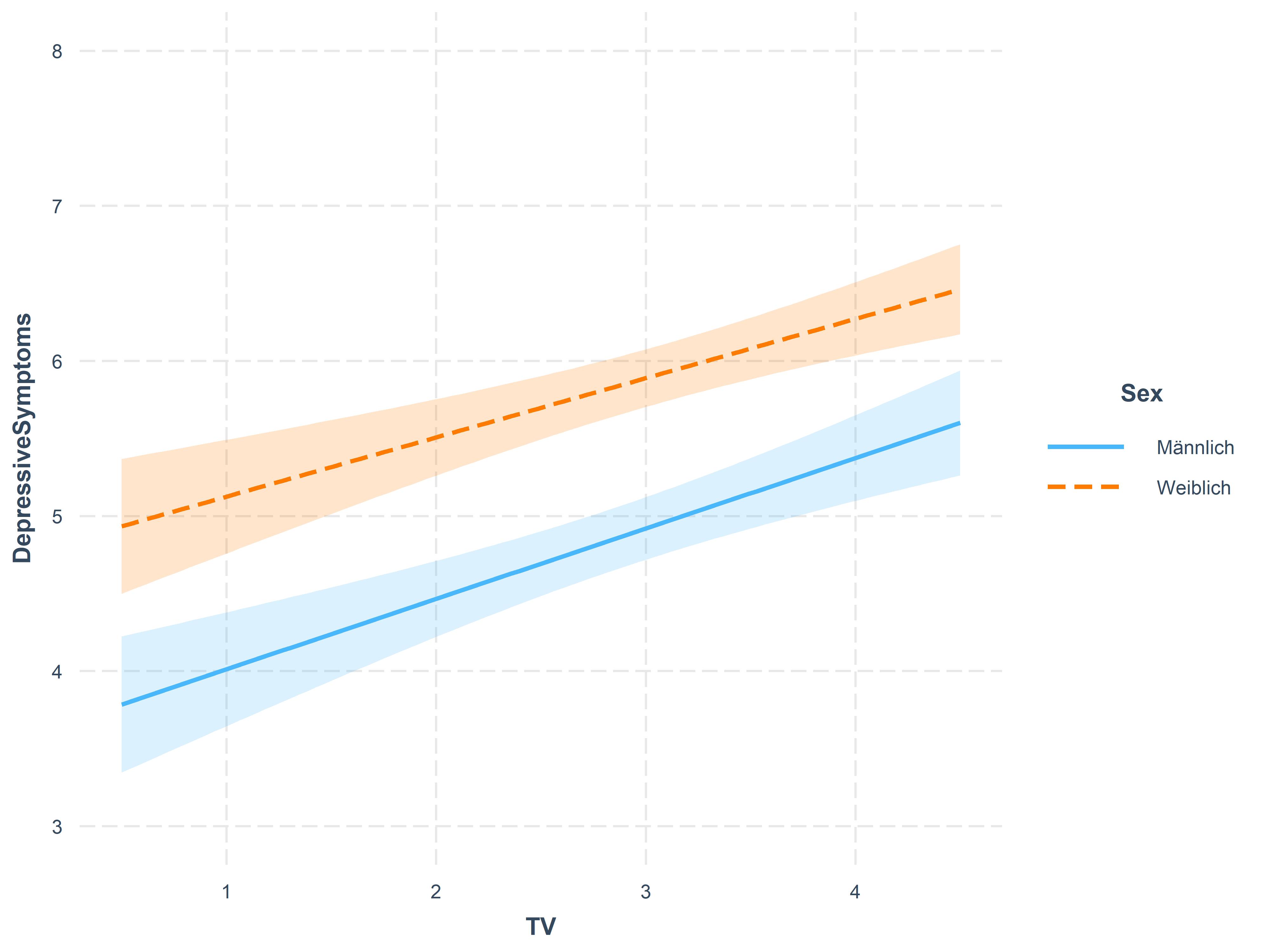

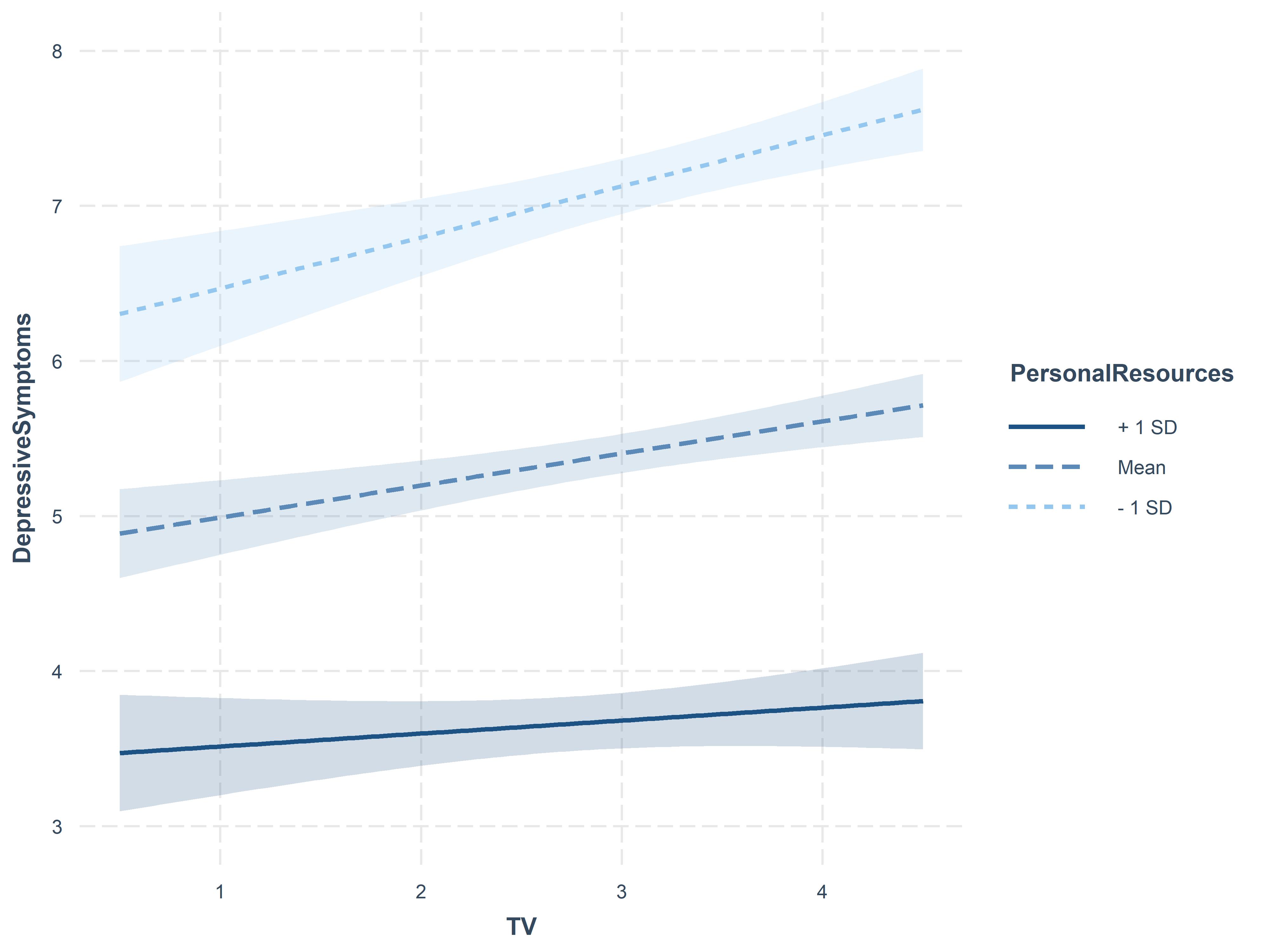

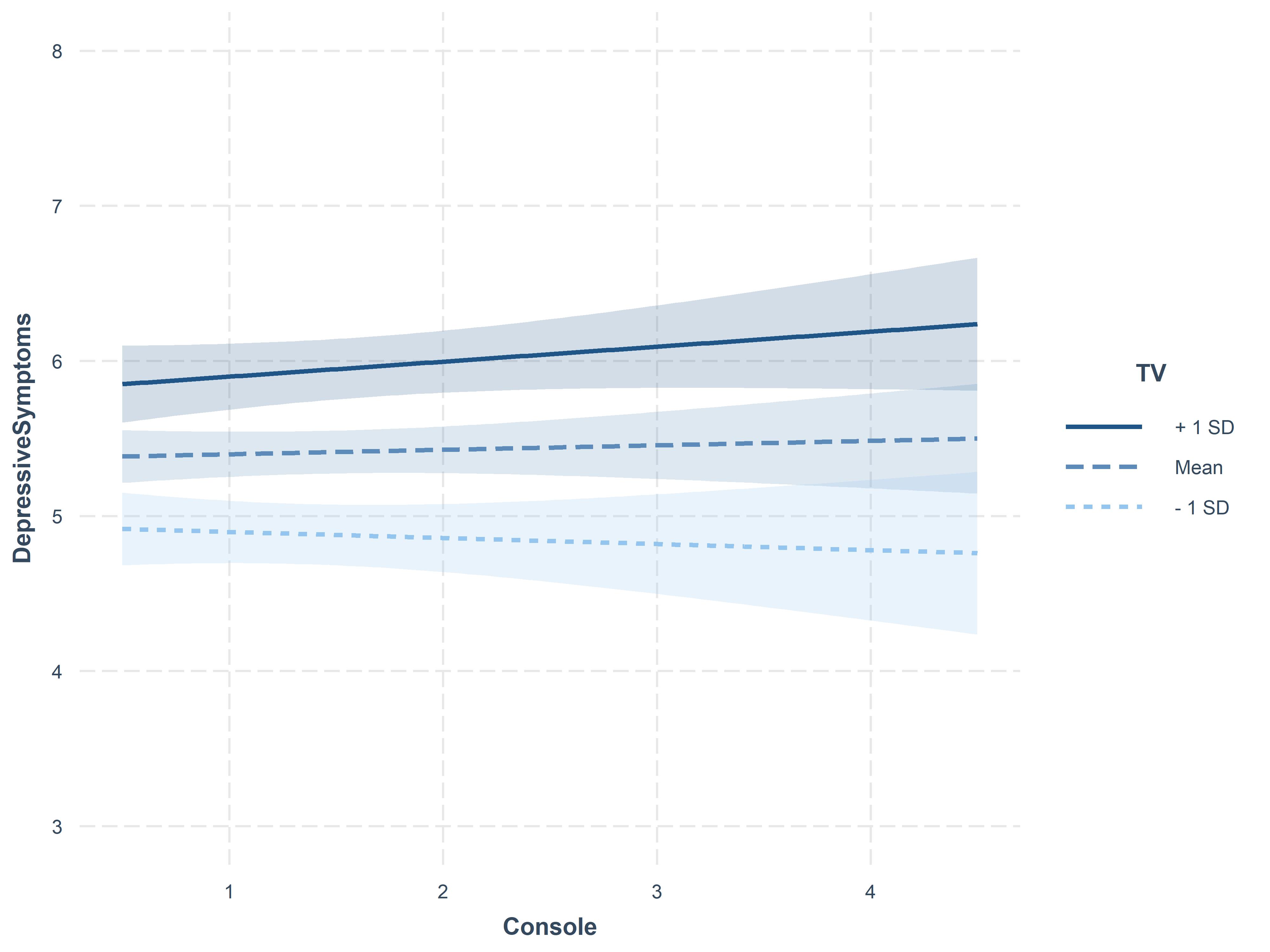


**Fig. A3**. Interaction plots showing simple slopes of health risk behaviours predicting depressive symptoms (min=0, max=25) for 1 SD below (19.04), 1 SD above (25.23) and at the mean level of age (*M*=22.14); for sex (46 % male); for 1 SD below (55.59), 1 SD above (83.89) and at the mean level of personal resources (*M*=69.74); for 1 SD below (1.75), 1 SD above (4.17) and at the mean level of TV (*M*=2.96). Coloured shading represent 95% CIs.


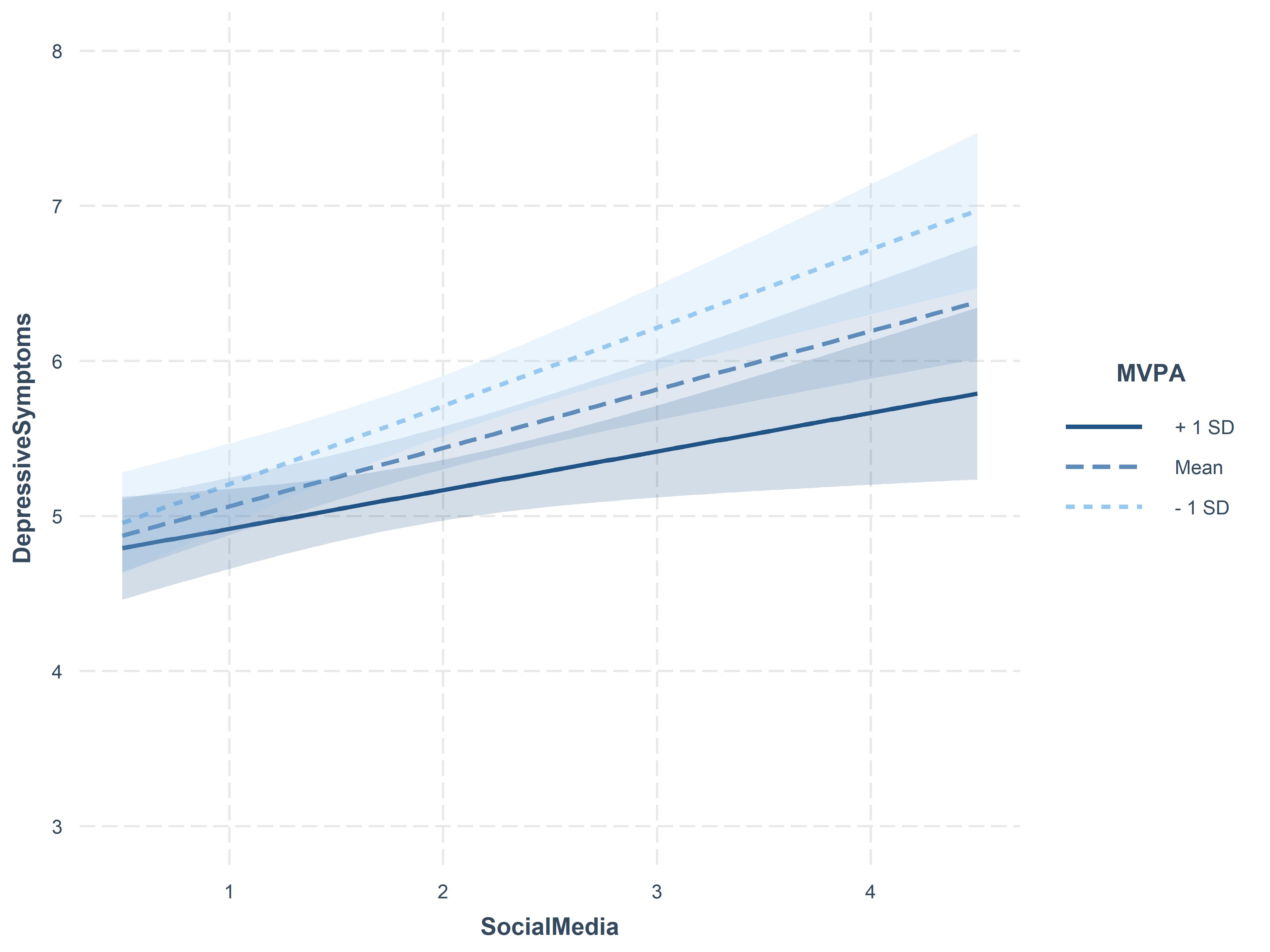

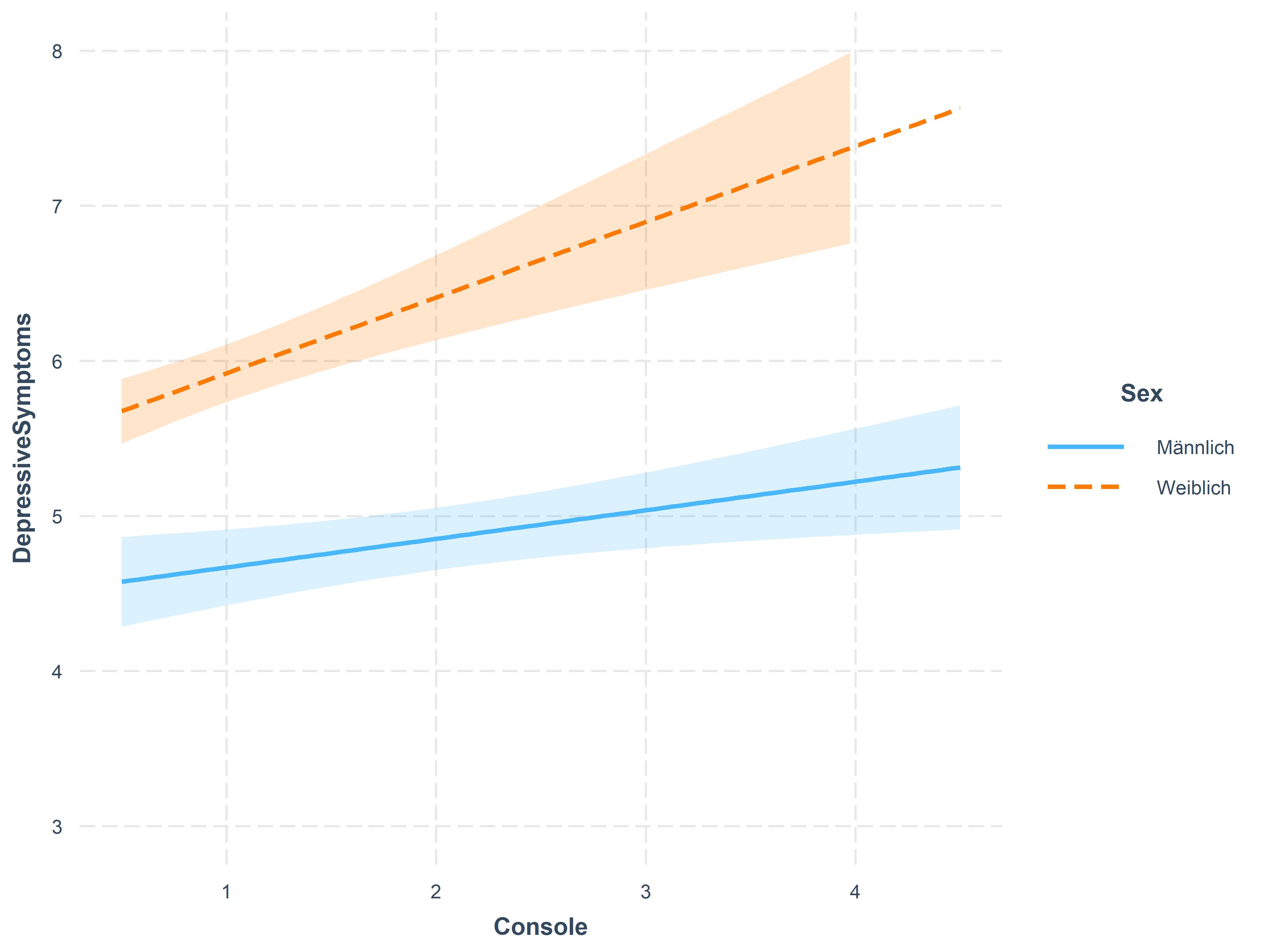


**Fig. A4**. Interaction plots showing simple slopes of health risk behaviours predicting depressive symptoms (min=0, max=25) for 1 SD below (25.79), 1 SD above (70.21) and at the mean level of MVPA (*M*=48.00); for sex (46 % male). Coloured shading represent 95% CIs.

**Table A1**

Simple slope analyses for significant interactions resulting from elastic net regression on depressive symptoms

| *Predictor* | *Moderator (1 SD below, 1 SD above and at the mean level)* | | *Estimate* | | *Std. Error* | *p* |
| --- | --- | --- | --- | --- | --- | --- |
| MVPA | Social Media ^a^ | .90 | -.006 | .01 | | .379 |
|  |  | 1.94 | -.012 | .00 | | .014 * |
|  |  | 2.98 | -.018 | .01 | | .012 * |
| PC ^a^ | Socioeconomic status | 8.45 | .419 | .12 | | <.001 *** |
|  |  | 12.12 | .547 | .09 | | <.001 *** |
|  |  | 15.79 | .674 | .13 | | <.001 *** |
|  | Education | 3.63 | .578 | .11 | | <.001 *** |
|  |  | 4.05 | .516 | .09 | | <.001 *** |
|  |  | 4.48 | .454 | .12 | | <.001 *** |
|  | Age | 19.04 | .721 | .12 | | <.001 *** |
|  |  | 22.14 | .508 | .09 | | <.001 *** |
|  |  | 25.23 | .295 | .12 | | .013 * |
|  | Personal Resources | 55.59 | .606 | .11 | | <.001 *** |
|  |  | 69.74 | .444 | .08 | | <.001 *** |
|  |  | 83.89 | .282 | .11 | | .009 ** |
| TV ^a^ | Age | 19.04 | .561 | .12 | | <.001 *** |
|  |  | 22.14 | .448 | .09 | | <.001 *** |
|  |  | 25.23 | .336 | .13 | | .009 ** |
|  | Sex | Male | .454 | .13 | | <.001 *** |
|  |  | Female | .382 | .12 | | .002 ** |
|  | Personal Resources | 55.59 | .330 | .12 | | .005 ** |
|  |  | 69.74 | .207 | .08 | | .011 * |
|  |  | 83.89 | .084 | .11 | | .466 |
| *Predictor* | *Moderator (1 SD below, 1 SD above and at the mean level)* | | *Estimate* | | *Std. Error* | *p* |
| TV ^a^ | Console ^a^ | .14 | .366 | .12 | | .003 ** |
|  |  | 1.45 | .439 | .09 | | <.001 *** |
|  |  | 2.76 | .513 | .12 | | <.001 *** |
| Console ^a^ | Sex | Male | .184 | .11 | | .088 |
|  |  | Female | .488 | .15 | | .001 ** |
| *Note*: MVPA = Moderate to Vigorous Physical Activity;  ^a^ Self-reported average daily consumption answered on a 6-point scale from 0 (“Not at all”), 1 (“Up to 1 hour”), 2  (“1 up to 2 hours”), 3 (“2 up to 3 hours”), to 4 (“3 up to 4 hours”), 5 (“More than 4 hours”);  ^b^ Transformed sum score ranging from 0 to 100 based on 8 items answered on a 5-point scale from 1 (“Never”)  to 5 (“Always”);  ^c^ Mean of two items for each dimension answered on a 5-point scale from 1 (“Disagree strongly”) to 5 (“Agree  strongly”). | | | | | | |
